# Evaluation of Remineralization of White Spot Lesions with Quercetin Nanoparticles: An *in vitro* Study

**DOI:** 10.4317/jced.62221

**Published:** 2025-02-01

**Authors:** Karthikeyan Subramani, Brian Decker, Kishore Chaudhry, Usha Subbiah, Eduardo G. Mota

**Affiliations:** 1 Roseman University of Health Sciences College of Dental Medicine, Henderson, Nevada, USA; 2 Sree Balaji Dental College and Hospital, Chennai, Tamilnadu, India; 3 Pontifical Catholic University of Rio Grande do Sul, Porto Alegre, Brazil

## Abstract

**Background:**

This study explored the synthesis of quercetin nanoparticles (QNPs) and evaluated the remineralization effect of QNPs on artificial white spot lesions on extracted human teeth.

**Material and Methods:**

QNPs were successfully synthesized, and their size was measured. Seventy-six extracted human molars were divided into 4 groups of n=19 to undergo a 10-day pH cycling protocol: 1000 ppm fluoride solution as aqueous NaF (group 1), 6.5% w/v quercetin microparticle solution (group 2), 4000 ppm QNP (group 3) and deionized water (group 4). Vickers microhardness tester, scanning electron microscopy-energy dispersive X-ray spectroscopy (SEM-EDX) and atomic force microscopy (AFM) were used to measure the surface microhardness (SMH), Ca:P ratio and surface roughness (Ra), respectively.

**Results:**

After remineralization, the SMH values were significantly different among all the experimental groups (*p*<0.001). The fluoride and QNP groups had significantly greater SMH values than the quercetin group. The AFM data showed a significant decrease, but the differences were not significant. The Ca:P values were significantly greater than those of the control in all 3 experimental groups, but the QNP and fluoride concentrations were significantly greater than those of quercetin. There were no significant differences between QNPs and fluoride according to any test.

**Conclusions:**

It can be concluded from the results of this study that QNPs have similar remineralization potential to fluoride and are more effective than quercetin.

** Key words:**White Spot Lesion, Remineralization, Quercetin, Nanoparticles, Orthodontic Treatment.

## Introduction

Patients undergoing orthodontic treatment tend to exhibit plaque buildup around brackets if they do not maintain good oral hygiene. Orthodontic elastomeric ligature ties and chains are more prone to dental plaque buildup. This often results in enamel demineralization and the development of white spot lesions (WSLs) on enamel surface around the brackets (1). WSLs are subsurface enamel porosities from carious demineralization that manifest clinically as a milky white opaque appearance of the enamel (2). The prevalence of WSLs in patients undergoing orthodontic treatment has been reported to be as high as 46% (3). WSLs are caused by an imbalance between the dynamic biological processes of demineralization and remineralization of enamel. These two biological processes depend on various factors, such as calcium (Ca2+), phosphate (PO4)3− and fluoride (F-) ions in saliva and plaque as well as the buffering capacity of saliva and oral hygiene (2). Natural remineralization of the surface of WSLs from salivary ions [Ca2+, (PO4)3−, F-] has very little effect on the esthetic appearance and structural properties of deeper WSLs. Current treatment options for WSLs include resin infiltration, microabrasion, vital bleaching, direct/indirect restorations, and the use of remineralizing agents (4,5). To aid in the remineralization of deeper WSLs, remineralizing agents such as casein phosphopeptide-amorphous calcium phosphate (CPP-ACP) (MI Paste®) (6) and fluoride supplements in the form of mouth rinses, gels or topical creams have been used (7,8). However, these remineralizing agents have certain limitations. Casein phosphopeptide-amorphous calcium phosphate (CPP-ACP) is associated with the milk-derived protein RECALDENT™, which is the active ingredient in MI Paste®. An 11-year-old girl with a milk allergy from California, USA, died due to an adverse allergic reaction to RECALDENT™ after her Dentist prescribed MI paste for WSL treatment (9). Fluoride treatment on enamel surface after the extensive WSLs can result in the formation of harder fluorapatite crystals on the enamel surface, leaving milky white discoloration of the WSLs to persist in the deeper layers of enamel. It was also reported that MI paste and fluoride varnish do not appear to be more effective than regular home care (brushing with fluoride toothpaste and flossing) for improving the appearance of WSLs (10). With these limitations of currently available remineralizing agents, newer biocompatible materials need to be explored for the treatment and remineralization of WSLs after orthodontic treatment.

**Figure 1:**
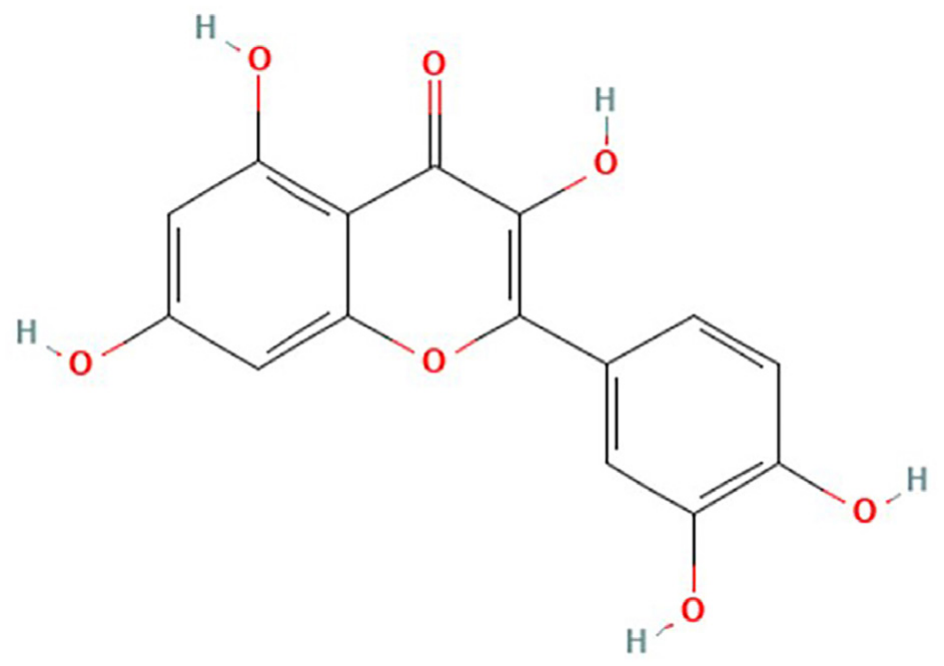
Chemical structure of Quercetin (20).

Various biomolecules, including proteins, peptides, nucleic acids, liposomes, carbohydrates and phosphorus-containing biomolecules, have been explored as biotemplates for biomimetic mineralization and for hydroxyapatite crystal formation from calcium and phosphate ions (11). The use of phytochemicals (plant chemicals) isolated from dietary plants could balance the oral flora, primarily *Streptococcus mutans*, which metabolizes sucrose to lactic acid that dissolves the hydroxyapatite crystals in enamel, causing demineralization, WSLs and eventual tooth decay (12). The investigation of phytochemicals for the remineralization of WSLs could lead to the development of novel approaches for safer and more natural alternatives to currently available remineralizing agents. Flavonoids are phytochemicals and plant pigments found in almost all fruits, vegetables, and beverages, such as tea and wine (13,14). Flavonoids are polyphenolic compounds that are composed of multiple phenol rings with hydroxyl groups (OH) attached to the phenol rings (15). Quercetin is a flavonoid found in vegetables (garlic, onion), fruits (apples, grapes, citrus fruits, berries) and plant based beverages (red wine, green tea). Quercetin is safer for human consumption and has antioxidant, antibacterial, anticancer, anti-inflammatory and cardio- and neuro-protective properties (13,14,16,17). Apart from these beneficial bioactive properties, quercetin can also interact with proteins such as collagen (18) and has been shown to increase bone mass and density (19). The chemical structure of quercetin is shown in Figure 1 (20). An earlier *in vitro* study showed that quercetin was effective at inhibiting demineralization and enhancing remineralization of artificial root caries lesions (21). In this study, three flavonoids (6.5% quercetin, 6.5% naringin and 6.5% proanthocyanidin) were tested in solution, and the remineralization effect was compared with that of a 1000 ppm fluoride solution. These three flavonoids had positive effects on artificial root caries remineralization, but the effect was less pronounced than that of 1000 ppm fluoride. As flavonoid microparticles (10-6 m) in solution form were used in this study on the dentine surface, the results of such a study need to be re-evaluated to explore whether the smaller nanoparticle (10-9 m) form of quercetin has superior remineralization potential of enamel. As the nanoparticles are much smaller than the particles at the microscale dimension, there is a possibility of a more deposition of nanoparticles in the deeper layers of WSLs of enamel and the hydroxyl group of the Quercetin can facilitate in the formation of hydroxyapatite crystals and remineralization.

Quercetin nanoparticles (QNPs) can be prepared from quercetin solution by anti-solvent precipitation under sonication (22). Due to the limitations of currently available remineralizing agents, evaluating the remineralization potential of naturally occurring bioactive compounds, such as quercetin, in nanoparticulate form is essential. Therefore, this *in vitro* study aimed to evaluate the remineralization potential of QNPs compared to that of fluoride and quercetin microparticle solutions. The null hypothesis tested in this study was that there was no significant difference between the effects of QNPs, quercetin microparticles and NaF solutions on the remineralization of WSLs.

## Material and Methods

-Specimen preparation and baseline surface microhardness evaluation

All chemical reagents used in this study were purchased from Sigma‒Aldrich (St. Louis, MO, USA). Seventy-six extracted permanent human molars which were originally extracted for clinical purposes from different Oral Surgery practices were collected. Since the patients needed these teeth extracted for clinical purposes, no other patient identifiers were collected/kept along with the teeth. IRB determined that this study did not meet the definition of human subject research, according to federal regulations and the need for informed consent for collection of teeth was waived by IRB. The collected teeth were stored in 0.1% thymol solution. All the collected teeth were examined under 4.5x magnification (Carson Optical, Ronkonkoma, NY, USA), and the teeth with any hypoplastic lesions, caries, cracks, WSLs, or stains were discarded. All soft and hard tissue debris was removed from the selected teeth and stored in 0.1% thymol solution for further use. Teeth were sectioned 1 mm below the cementoenamel junction with a slow-speed diamond disc (Kerr Dental, Brea, CA, USA) under running deionized water, and the roots were discarded. 3D-printed cylindrical resin molds were fabricated with a 3D printer (Stratasys, Tucson, AZ, USA), and the teeth were placed in molds with their buccal surface adhered to double-sided adhesive tape on a workbench to ensure that the buccal surfaces remained exposed and parallel to the horizontal plane of the molds. Polymethyl methacrylate resin (Miami Dental Supply, Miami, FL, USA) was poured into the molds, which were subsequently cured to provide a sTable mount. The exposed buccal surfaces were sequentially polished using progressive grit polishing discs (Kerr Dental, Brea, CA, USA) to ensure that the smooth enamel surface was free of micro imperfections. A 5 mm × 5 mm window of exposed enamel was created in the middle of the buccal surface by covering the rest of the buccal surface with acid-resistant nail varnish (OPI, New York, NY, USA).

Forty tooth samples were used for surface microhardness measurements, and 36 samples were subjected to atomic force microscopy (AFM) to measure the surface roughness average (Ra) and to scanning electron microscopy-energy dispersive X-ray spectroscopy (SEM-EDX) to analyze the chemical composition (calcium:phosphorus ratio) of the enamel surface. Before artificial WSL formation, the baseline enamel surface microhardness was measured on 40 tooth samples using a Vickers microhardness tester (Metal Testers, New York, NY, USA). Indentations were made at 3 points spaced 500 μm from each other at the center of the exposed window using a 100 g load for 10 seconds. The measurements were averaged, and samples with a Vickers microhardness > 430 or < 340 were excluded from the study.

-Synthesis of quercetin nanoparticles and remineralizing solutions

QNPs were synthesized *in vitro* by the antisolvent precipitation method (22). Absolute ethyl alcohol against water was used as the solvent and antisolvent at a 1:20 ratio. QNPs were then synthesized by dissolving 100 mg of quercetin in 5 mL of absolute ethyl alcohol. The resulting solution was added to 100 mL of 0.15% (w/v) aqueous solution containing 4:1 w/w hydroxypropyl methylcellulose (HPMC) and sodium lauryl sulfate (SLS). This solution was cooled to 8°C in an ice-water bath and sonicated. The precipitation rate was controlled throughout the process by maintaining the temperature below 8°C using an ice-water bath. The particle size was reduced with an ultrasonic probe sonicator (Benchmark Scientific, Sayreville, NJ, USA) at an ultrasonic power input of 300 W for 10 min. The suspension was placed in a ROTA evaporator (Heidolph, Wood Dale, IL, USA) at 40°C for 10 min to remove the organic solvent. The QNP suspensions were further homogenized for 30 min to obtain the final preparation. The homogenized suspension was evaluated for particle size distribution with a nanoparticle size analyzer (Zetasizer Nano, Malvern Panalytical, Malvern, UK) and further confirmed using a scanning electron microscope (SEM; JSM-6700F, JEOL Ltd., Tokyo, Japan). A 6.5% (w/v) solution of quercetin in phosphate buffer (0.025 M KH2PO4, 0.025 M K2HPO4, pH 7.4) was also used in this study to evaluate whether there was a difference between the remineralization potential of the quercetin nanoparticles and the quercetin solution. Sodium fluoride (NaF) solution (1000 ppm) was used as a positive control, and deionized water was used as a negative control. The test groups were designated as follows.

Group 1 – Sodium fluoride (NaF) solution

Group 2 – Quercetin microparticle solution (referred as Quercetin in this manuscript)

Group 3 – Quercetin nanoparticle (QNP) solution

Group 4 - Deionized water

-Artificial White Spot Lesion Formation

Artificial WSLs were created by immersing the selected tooth samples in a demineralizing solution (50 mM acetic acid, 10 mM NaH2PO4–2H2O, 2.2 mM CaCl2–2H2O, 100 mM NaCl, 1 ppm NaF, 5 mM NaN3), and the pH was adjusted to 4.5 using 1 M NaOH solution at 37°C for 4 days under continuous, low-speed magnetic stirring (100 rpm) (Benchmark Scientific, Sayreville, NJ, USA) (23,24). Thereafter, the samples were rinsed with deionized water spray for 15 s followed by ultrasonication in deionized water 3 times (5 min per wash) to terminate demineralization. Following demineralization, surface microhardness measurements were taken on 40 tooth samples to obtain post-demineralization data using the protocol described previously.

-*In vitro* remineralization of WSLs

The tooth samples were then subjected to 10 days of pH cycling for *in vitro* remineralization of artificial WSLs. All the solutions were maintained at 37°C to simulate the oral environment. The samples in each group were pH cycled through the respective treatment solution for 10 minutes, followed by demineralization with acidic buffer for 30 minutes (50 mM acetic acid, 10 mM NaH2PO4–2H2O, 2.2 mM CaCl2–2H2O, 100 mM NaCl, 1 ppm NaF, 5 mM NaN3, pH 4.5) and remineralization with neutral buffer for 10 minutes (20 mM HEPES; 2.25 mM CaCl2.2H2O; 1.35 mM KH2PO4; 130 mM KCl, pH 7.0) (21). When the solutions were switched between, the samples were copiously irrigated for 15 seconds using deionized water. The pH of all the solutions was checked daily, and the solutions were confirmed to be stable. Six separate demineralization–remineralization cycles were performed each day for 10 days. All the samples were stored overnight in neutral buffer at 37°C.

-Calcium: Phosphorus Ratio Analysis and Surface Roughness Evaluation

Atomic force microscopy (AFM) was used to measure the surface roughness average (Ra), and scanning electron microscopy (SEM)-energy dispersive X-ray spectroscopy (SEM-EDX) was utilized for chemical composition analysis (calcium:phosphorus ratio) of 36 samples (12 samples before demineralization, 12 samples after demineralization and 12 samples after remineralization). AFM analysis was first performed, followed by SEM-EDX analysis, on the same samples. For AFM analysis, images were acquired using an XE-70 PSIA AFM (Park Systems, South Korea) in noncontact mode using PPP-NCHR AFM probes (NANOSENSORS, Switzerland) at multiple locations with scan sizes of 5x5, 10x10, 20x20, 40x40, 60x60, 80x80 and 100x100 µm2, of which the 60x60 µm2 images were used for further analysis. Line profiles of pit features and their surrounding areas were extracted and analyzed using Park Systems XEI Imaging and OriginLab Data Analysis Software.

For SEM-EDX analysis, the enamel specimens were cut with low-speed diamond discs (Buehler, U.S.A.) and refined with carbide burs (Komet, U.S.A.) to 4 mm × 3 mm × 1 mm pieces. The specimens were then gold-coated with a Cressington Sputter Coater 108 Auto (Cressington Scientific Instruments, United Kingdom) to alleviate charging effects. SEM-EDX was carried out using a JEOL JSM-5600 Scanning Electron Microscope (JEOL, U.S.A.) at 15 kV (aperture 2, spot size 30, working distance 15 mm). Elemental X-ray maps were collected in a 256 × 256 pixel matrix with 200 scan frames using the INCA Microanalysis Suite (Oxford Instruments, United Kingdom). A 1 mm × 1 mm area analysis was performed on each specimen, and the calcium-to-phosphorus (Ca:P) ratio was calculated. Due to the gold coating, we could not evaluate each specimen longitudinally. Thus, specimens were taken from multiple teeth to allow for a better determination of the Ca:P ratio.

Evaluation of remineralization and Statistical analysis

The baseline, post-demineralization, and post-remineralization data were analyzed with a predetermined significance level of 0.05 with one-way ANOVA and a Tukey HSD post hoc test using SPSS version 29.0 to analyze the changes in surface microhardness (SMH), surface roughness average (Ra) and chemical composition [calcium:phosphorus (Ca:P) ratio].

## Results

-Quercetin nanoparticle analysis

**Figure 2:**
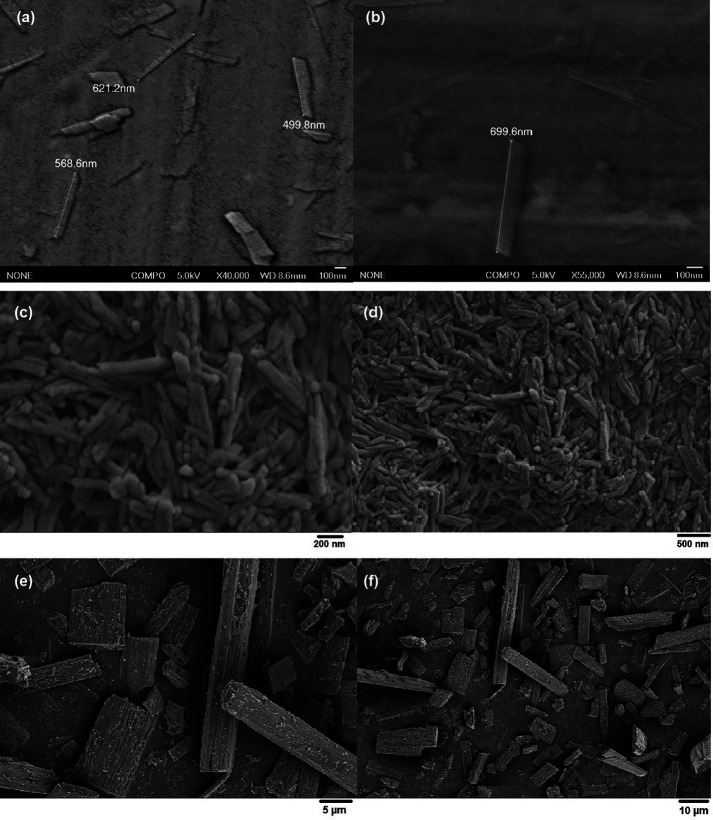
Scanning electron microscopy image of QNPs spread on a glass coverslip (a) at 40,000x magnification, (b) at 55,000x magnification, (c) at 100,000x magnification, (d) at 50,000x magnification, (e) at 5000x magnification and (f) at 2,000x magnification.

The average size of the QNPs in suspension was measured with a Zetasizer Nanoparticle Analyzer and was 678.9 nm. Further SEM analysis of the QNPs revealed that the QNPs were rod shaped with lengths ranging from approximately 500 nm to 700 nm and widths of approximately 162 nm (Fig. 2a-d). The quercetin microparticles were approximately 5 µm to 50 µm in length and 5 µm to 10 µm in width (Fig. 2e,f).

-Evaluation of Surface Microhardness

 Table 1 shows the mean ± SD for surface microhardness measured at baseline, after demineralization (WSL formation) and after remineralization. The baseline microhardness [Vickers hardness number (VHN)] of group 1 was 322.39 ± 6.42, that of group 2 was 321.38 ± 3.54, that of group 3 was 321.04 ± 4.73, and that of group 4 was 322.50 ± 2.74. The microhardness after demineralization was 157.42 ± 6.14 in group 1, 158.11 ± 5.52 in group 2, 157.98 ± 5.89 in group 3 and 159.33 ± 4.2 in group 4. The microhardness after remineralization was 242.85 ± 10.63 in group 1, 216.50 ± 8.20 in group 2, 242.40 ± 7.17 in group 3 and 163.56 ± 4.6 in group 4.

-Calcium: Phosphorus Ratio Analysis and Surface Roughness Evaluation

**Figure 3:**
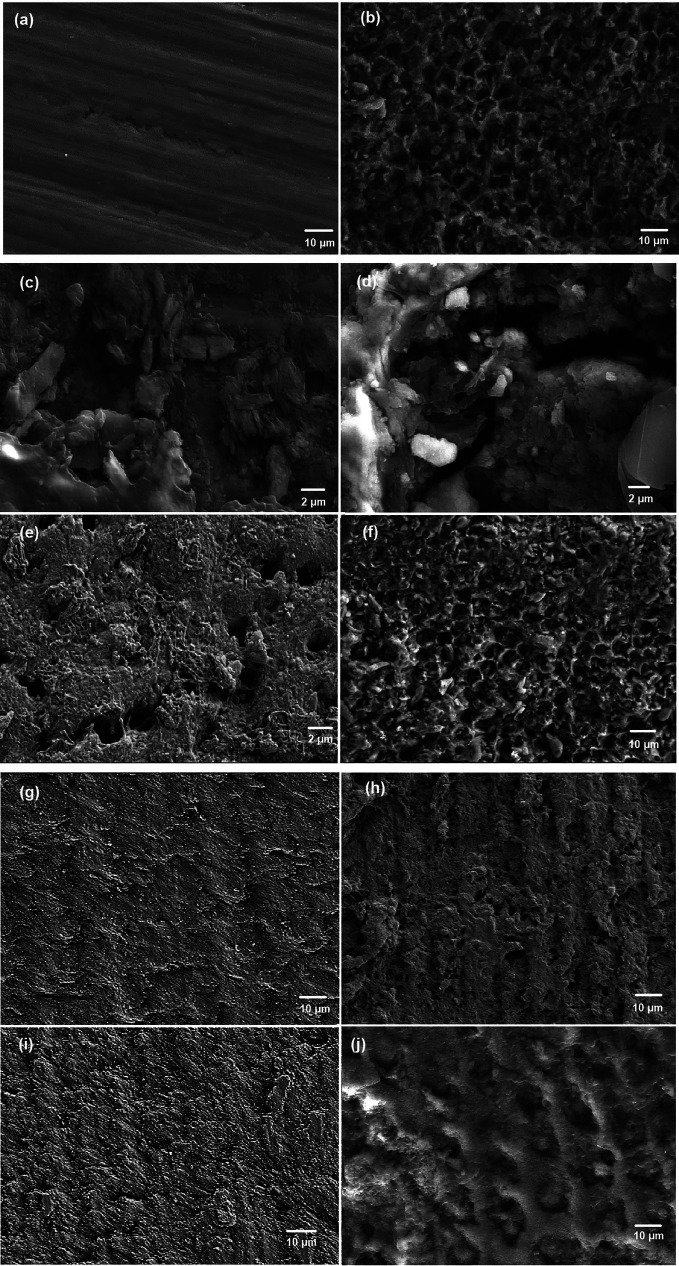
SEM images of (a) the enamel surface at baseline and (b) after demineralization. SEM images of particle deposition after 1 round of exposure to (c) fluoride solution, (d) quercetin solution, (d) QNP solution, and (f) deionized water; SEM images after remineralization with (g) fluoride solution, (h) quercetin solution, (i) QNP solution and (j) deionized water.

SEM-EDX elemental analysis of the Ca:P ratio (mean ± SD) at baseline, after demineralization and remineralization is shown in  Table 2. The baseline Ca:P ratio of group 1 was 2.08 ± 0.12, that of group 2 was 2.08 ± 0.1, that of group 3 was 2.08 ± 0.02, and that of group 4 was 2.08 ± 0.05. The Ca:P ratio after demineralization in group 1 was 1.35 ± 0.86, that in group 2 was 1.36 ± 0.75, that in group 3 was 1.35 ± 0.1, and that in group 4 was 1.35 ± 0.79. The Ca:P ratio after remineralization in group 1 was 1.95 ± 0.01, that in group 2 was 1.56 ± 0.04, that in group 3 was 1.96 ± 0.02, and that in group 4 was 1.38 ± 0.2. SEM image analysis revealed a smoother enamel surface at baseline (Fig. 3a) and a rougher enamel surface after demineralization with enamel rods exposed (Fig. 3b). SEM images of the particle deposition after 1 round of exposure to fluoride solution (Fig. 3c) and quercetin solution revealed inhomogeneous particle deposition (Fig. 3d). SEM images of the samples analyzed after 1 round of exposure to the QNP solution revealed QNP deposition on the enamel surface (Fig. 3e) and no particle deposition after 1 round of exposure to deionized water (Fig. 3f). SEM images after remineralization with fluoride solution (Fig. 3g) and QNP solution (Fig. 3i) showed uniformly remineralized enamel surfaces. SEM images after remineralization with quercetin solution showed an inhomogeneous remineralized enamel surface (Fig. 3h), and those with deionized water had a rougher enamel surface with a demineralized appearance (Fig. 3j).

**Figure 4:**
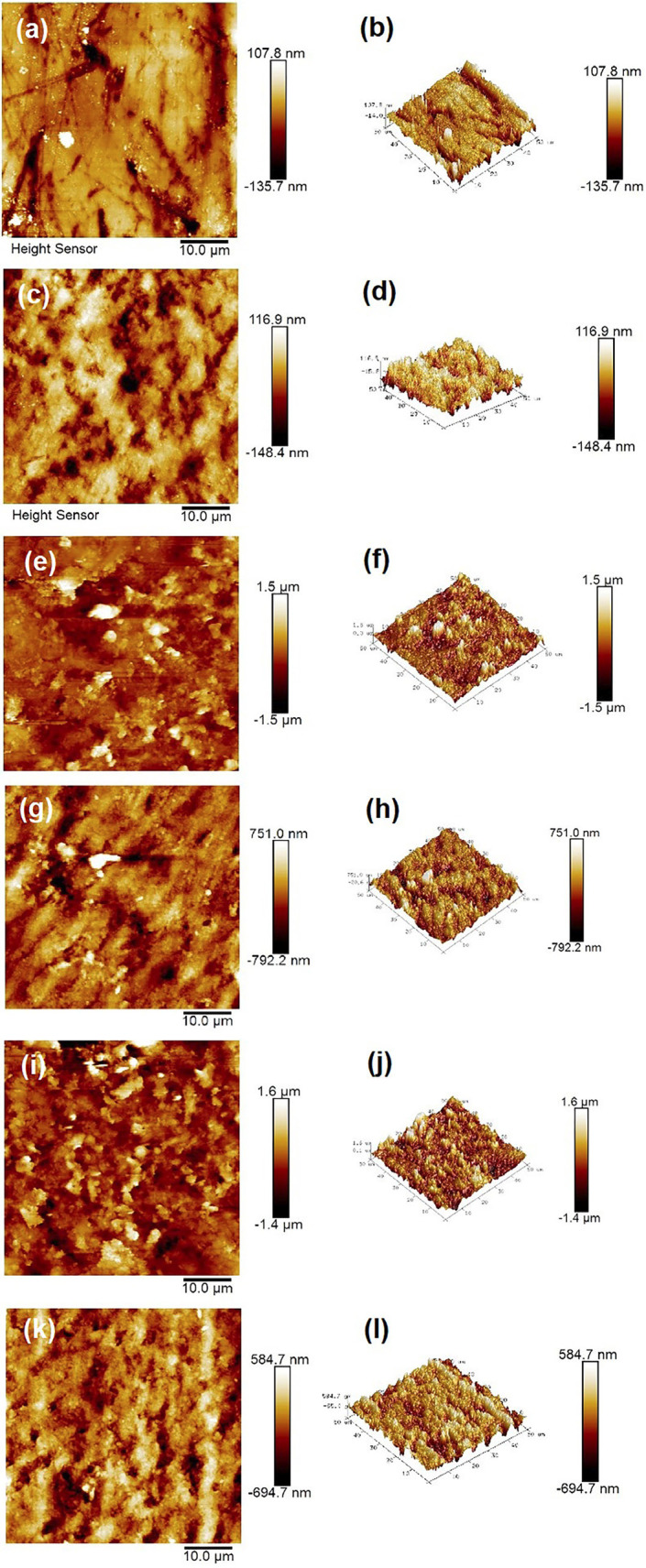
(a,b) 2D and 3D AFM images of the enamel surface at baseline; (c,d) demineralized enamel; 2D and 3D AFM images of the remineralized enamel surface treated with (e,f) fluoride solution, (g,h) quercetin solution, (i,j) QNP solution and (k,l) deionized water.

The AFM results of the surface roughness average (Ra) in nm (mean ± SD) measured at baseline, after demineralization and after remineralization are shown in  Table 3. The baseline Ra of group 1 was 24.3 ± 4.41, that of group 2 was 24.63 ± 2.95, that of group 3 was 24.83 ± 2.77, and that of group 4 was 24.73 ± 2.13. The Ra after demineralization in group 1 was 439.87 ± 21.45, that in group 2 was 439.53 ± 13.96, and that in group 3 was 439. 7 ± 14.92, and that of group 4 was 439.67 ± 16.95. The enamel surface was roughest after demineralization (Fig. 4a, b) compared to the baseline surface, which was smoother (Fig. 4c, d). The Ra after remineralization was 269.83 ± 16.54 in group 1, 268.73 ± 11.37 in group 2, 270.53 ± 4.07 in group 3 and 398.6 ± 19.03 in group 4. The AFM images of the enamel surface after remineralization with fluoride (Fig. 4e, f) and QNPs (Fig. 4i, j) revealed a slightly rougher surface, followed by those obtained with quercetin (Fig. 4g, h) and deionized water (Fig. 4k, l).

## Discussion

Quercetin is a yellow-colored pigment (25). Even though the Quercetin and QNP solutions used in this study were yellow colored, remineralization of artificial WSLs with these solutions did not cause any discoloration of the enamel samples. Surface microhardness evaluation is one of the mostly widely used non-destructive tests to measure the mechanical properties of a substrate material. The surface is indented with a square-diamond tip with a certain load for a period of time and the indentation area is measured to calculate the surface microhardness of the sample (26). As expected, all the groups exhibited a statistically significant reduction in surface microhardness after demineralization ( Table 1). The surface microhardness levels were similar in all the experimental groups before (*p*=0.858) and after demineralization (*p*=0.885) which showed the uniformity of artificial WSL formation on all samples after demineralization. However, the surface microhardness was significantly different among the groups after remineralization (*p*<0.001). The control group (treated with deionized water) exhibited a very small increase in surface microhardness from demineralized teeth compared to that of all the other three experimental groups, which was significantly lower than that of all the other groups (*p*<0.001 from all three experimental groups). This showed that the DI water had very minimal effect on restoring surface microhardness. The quercetin-treated group exhibited greater remineralization than did the control group but significantly less remineralization than did the other two experimental groups did (*p*<0.001 compared with all the other groups). The remineralization of the fluoride-treated and QNP-treated groups was significantly greater than that of the quercetin-treated and control groups. However, the difference between these two groups (fluoride and QNP) was not statistically significant (*p*=0.999), indicating that these two groups were equally effective. This showed that the QNPs and fluoride had similar effect in restoring the surface microhardness of enamel and higher than Quercetin. However, none of the demineralized teeth were able to achieve their baseline microhardness level in any group.

SEM-EDX is a quantitative X-ray microanalytical technique which uses X-rays to evaluate the elemental chemical composition of a surface and is used with SEM to magnify and scan a sample area (27,28). In this study, SEM-EDX was used to evaluate changes in elemental chemical composition of Ca:P ratio at baseline, after demineralization and after remineralization. In all four groups, the SEM-EDX measurements of the Ca:P ratio decreased after demineralization and increased after remineralization. However, even after remineralization, the value did not return to the baseline level for any tooth. The changes associated with the demineralization-remineralization process were significantly different only for the QNP group ( Table 2), and the values after demineralization were significantly different from those at baseline and for the remineralization groups (*p* <0.001 for both comparisons). The change in Ca:P ratios did not significantly differ between the experimental groups at baseline (*p*=1.0) or after demineralization (*p*=1.0). After remineralization, the values were similar in the fluoride and QNP groups (*p*=1.0), suggesting similar improvements. The values in the fluoride and QNP groups were significantly greater than those in the quercetin group (*p*=0.007 and <0.001, respectively) and the deionized water group (<0.001 for both comparisons). This showed that QNPs and fluoride had similar potential in restoring Ca:P ratio of enamel surface following remineralization and higher than the Quercetin solution.

The AFM measurements of Ra were similar in all four experimental groups at baseline (*p*=0.997) and after demineralization (*p*=1.0). After remineralization, the AFM values were significantly lower in all three experimental groups than in the control group (*p*<0.001 for all comparisons). An earlier *in vitro* study evaluated the remineralization of artificial root caries on dentin specimens and used three flavonoids in solution form (21). This study revealed that flavonoids (6.5% quercetin, 6.5% naringin and 6.5% proanthocyanidin) had positive effects on artificial root (dentine) caries remineralization but had weaker effects than 1000 ppm fluoride. Our study focused on fabricating QNPs and evaluating their remineralization effects on WSLs on enamel surface and compared it to 6.5% quercetin and 1000 ppm fluoride. Our results demonstrated that QNPs can be made from quercetin microparticles, and the SMH and SEM-EDX analyses showed that the QNPs had greater remineralization potential than the quercetin microparticle solution and that the remineralization efficiency of the QNPs was comparable to that of fluoride. One possible explanation for the superior remineralization potential of QNPs compared to microparticles may be their smaller size. Smaller particles can penetrate deeper into the enamel at higher concentrations and act as nucleation sites, providing more effective mineral deposition. Quercetin, a polyphenol, is composed of multiple phenol rings with hydroxyl groups. Hydroxyl groups play a crucial role during remineralization and in the formation of hydroxyapatite. Earlier studies have shown that Quercetin can interact with collagen and increase bone mass and density (18,19). The smaller size of QNPs and their synergistic effect on hydroxyapatite crystal formation and interaction with collagen protein to promote remineralization can explain the results of this study.

The results of our *in vitro* study have certain limitations. First, *in vitro* studies lack the complexity and diversity of oral biofilms that exist *in vivo*. Extracted teeth lack blood supply and are more prone to dehydration, which may influence the outcomes of such *in vitro* studies. The present study was limited by the use of surface microhardness evaluation, SEM-EDX, and AFM to quantify remineralization. Additional studies could use additional complementary techniques, such as micro-CT, to confirm the findings of this study to evaluate enamel remineralization in deeper layers. Quercetin powder is currently available as a dietary supplement in Tablet form and is safer for human consumption (29,30). Previous studies have shown that it has antioxidant, antibacterial, anticancer, anti-inflammatory and cardio- and neuro-protective properties (13,14,17,25). The cytotoxicity of QNPs used in this study has not been evaluated. Evaluation of cytotoxicity would further support the potential clinical use of QNPs towards remineralization of WSLs. It has been shown in a recent study that green synthesized silver nanoparticles (AgNPs) loaded with QNPs exhibit inhibition of *in vitro* bacterial biofilms (31). Different combinations of QNPs and antibacterial agents can also help in reduction of plaque formation during orthodontic treatment and in the treatment of WSLs. Considering the limitations of currently available treatment modalities of WSL treatment, use of naturally occurring flavonoid like Quercetin in nanoparticulate form offers promising clinical applications, which needs to be explored further.

## Conclusions

Within the limitations of this *in vitro* study, it can be concluded that QNPs have greater remineralization potential than quercetin solution and is similar to fluoride solution.

## Figures and Tables

**Table 1:** Surface microhardness measurements (n=10/group) at baseline, after demineralization (WSL formation) and after remineralization are shown as the Vickers hardness number (VHN) (mean, SD). The measurements are an average of three indentations spaced 500 μm apart from each other in each enamel sample.

Groups	Mean	Std. Deviation	P (t test)		
			B* Vs D*	D* Vs R*	B* Vs R*
Fluoride					
Baseline	322.39	6.42	<0.001		
After Demineralization	157.42	6.14			<0.001
After Remineralization	242.85	10.63		<0.001	
	P (ANOVA) <0.001				
Quercetin					
Baseline	321.38	3.54	<0.001		
After Demineralization	158.11	5.52			<0.001
After Remineralization	216.50	8.20		<0.001	
	P (ANOVA) <0.001				
Quercetin Nanoparticles					
Baseline	321.04	4.73	<0.001		
After Demineralization	157.98	5.89			<0.001
After Remineralization	242.40	7.17		<0.001
	P (ANOVA) <0.001				
Deionized Water					
Baseline	322.50	2.74	<0.001		
After Demineralization	159.33	4.20			<0.001
After Remineralization	163.56	4.60		0.058	
	P (ANOVA) <0.001				

* B= Baseline; D=Demineralized Teeth; R=Remineralized Teeth.

**Table 2:** SEM-EDX measurements of the calcium-to-phosphorus (Ca:P) ratio (mean, SD) of the enamel surface were performed on 36 samples (n=3/group) at baseline, after demineralization (WSL formation) and after remineralization.

Groups	Mean	Std. Deviation	P (t test)		
			B* Vs D*	D* Vs R*	B* Vs R*
Fluoride					
Baseline	2.08	0.12	N/A		
After Demineralization	1.35	0.86			N/A
After Remineralization	1.95	0.01		N/A	
	P (ANOVA) =0.243				
Quercetin					
Baseline	2.08	0.10	N/A		
After Demineralization	1.36	0.75			N/A
After Remineralization	1.56	0.04		N/A	
	P (ANOVA) =0.192				
Quercetin Nanoparticles					
Baseline	2.08	0.02	<0.001		
After Demineralization	1.35	0.10		-	0.096
After Remineralization	1.96	0.02		<0.001	
	P (ANOVA) <0.001				
Deionized Water					
Baseline	2.08	0.05	N/A		
After Demineralization	1.35	0.79			N/A
After Remineralization	1.38	0.20		N/A	
	P (ANOVA) =0.175				

* B= Baseline; D=Demineralized Teeth; R=Remineralized Teeth.

**Table 3:** AFM measurement of the surface roughness average (Ra) in nm (mean, SD) measured in a 50x50 µm area of the enamel surface on 36 samples (n=3/group).

Groups	Mean	Std. Deviation	P (t test)		
			B* Vs D*	D* Vs R*	B* Vs R*
Fluoride					
Baseline	24.30	4.41	<0.001		
After Demineralization	439.87	21.45			<0.001
After Remineralization	269.83	16.54		<0.001	
	P (ANOVA) <0.001				
Quercetin					
Baseline	24.63	2.95	<0.001		
After Demineralization	439.53	13.96			<0.001
After Remineralization	268.73	11.37		<0.001	
	P (ANOVA) <0.001				
Quercetin Nanoparticles					
Baseline	24.83	2.77	<0.001		
After Demineralization	439.70	14.92			<0.001
After Remineralization	270.53	4.07		<0.001	
	P (ANOVA) <0.001				
Deionized Water					
Baseline	24.73	2.1362	<0.001		
After Demineralization	439.67	16.9542			<0.001
After Remineralization	398.60	19.0337		0.032	
	P (ANOVA) <0.001				

* B= Baseline; D=Demineralized Teeth; R=Remineralized Teeth.

## Data Availability

The datasets used and/or analyzed during the current study are available from the corresponding author.
